# Conjugation of Short Oligopeptides to a Second-Generation Polyamidoamine Dendrimer Shows Antibacterial Activity

**DOI:** 10.3390/pharmaceutics15031005

**Published:** 2023-03-21

**Authors:** Namyoung Kang, Le Thi Thuy, Viet Dongquoc, Joon Sig Choi

**Affiliations:** 1 Department of Biochemistry, Chungnam National University, 99 Daehak-ro, Yuseong-gu, Daejeon 34134, Republic of Korea; 2 Department of Materials Science and Engineering, Chungnam National University, 99 Daehak-ro, Yuseong-gu, Daejeon 34134, Republic of Korea

**Keywords:** polyamidoamine dendrimer, oligopeptide, cytotoxicity, antibacterial effect, cell membrane

## Abstract

The growing evolution of bacterial resistance to antibiotics represents a global issue that not only impacts healthcare systems but also political and economic processes. This necessitates the development of novel antibacterial agents. Antimicrobial peptides have shown promise in this regard. Thus, in this study, a new functional polymer was synthesized by joining a short oligopeptide sequence (Phe-Lys-Phe-Leu, FKFL) to the surface of a second-generation polyamidoamine (G2 PAMAM) dendrimer as an antibacterial component. This method of synthesis proved simple and resulted in a high conjugation yield of the product FKFL-G2. To determine its antibacterial potential, FKFL-G2 was subsequently analyzed via mass spectrometry, a cytotoxicity assay, bacterial growth assay, colony-forming unit assay, membrane permeabilization assay, transmission electron microscopy, and biofilm formation assay. FKFL-G2 was found to exhibit low toxicity to noncancerous NIH3T3 cells. Additionally, FKFL-G2 had an antibacterial effect on *Escherichia coli* and *Staphylococcus aureus* strains by interacting with and disrupting the bacterial cell membrane. Based on these findings, FKFL-G2 shows promise as a potential antibacterial agent.

## 1. Introduction

Bacterial resistance to antibiotics has become a serious issue, resulting in increasing difficulty in treating various infections and associated economic impacts [1,2]. This public health crisis has been the impetus for the development and improvement of new antibacterial agents, which differ considerably from conventional antibiotics [3]. In this context, synthesized chemical agents have attracted particular attention.

Polyamidoamine (PAMAM) dendrimers have become the most widely studied dendrimers for a range of biomedical applications, such as gene or drug delivery [4,5,6,7,8,9]. Their unique, highly branched, and globular structures enable them to function as a multivalent biocide or nanoscale antimicrobial drug carrier [10]. The homogeneous branching of dendrimers results in a high surface area-to-volume ratio, leading to high reactivity with microorganisms in vivo, such as in the cell membrane [11]. A previous study indicated that the generation 2 (G2) PAMAM can increase cell membrane permeability [12]. Additionally, the highly localized and densely packed functional groups on the surface of the dendrimer can be used to improve the targeting efficacy or conjugate targeting ligands to a variety of mammalian and microorganism receptors [13,14,15]. One of the advantages of such PAMAM dendrimer derivatives is that they can be synthesized simply, on a large scale, and at high purity [16]. Although their antibacterial activity has historically not been extensively investigated, PAMAM dendrimers have recently attracted the attention of researchers in the preparation of antibacterial agents. Calabretta et al. demonstrated the antimicrobial effect of native PAMAM dendrimers on ocular pathogens [17]. They found that the amine-terminated G5 PAMAM dendrimer was more toxic to *Pseudomonas aeruginosa* (minimum inhibitory concentration (MIC_50_) = 1.5 ± 0.1 μg/mL) than *Staphylococcus aureus* (MIC_50_ = 20.8 ± 3.4 μg/mL). The partial coating of the amine groups with polyethylene glycol (PEG) decreased G5 PAMAM toxicity against human corneal epithelial cells whilst retaining a high toxicity against *P. aeruginosa* (MIC_50_ = 0.9 ± 0.1 μg/mL). The PEGylation of G5 PAMAM resulted in a further reduction in toxicity against Gram-positive *S. aureus* [18]. Furthermore, they reported that, even though G5-PAMAM-NH_2_ has a greater localization of amines at its surface compared to generation 3 (G3) PAMAM, this does not translate to an increased antibacterial efficacy. Other studies have focused on the impact that native PAMAM dendrimers have on the antibacterial properties of PAMAM or antibiotics [19,20,21,22]. The G2 PAMAM dendrimer can function as an antibiotic supporter to improve its effectiveness [23]. The PAMAM dendrimer can also form a complex with TiO_2_ or copper as a nano-compound to inhibit bacterial growth [24]. Additionally, a bio-inspired, peptide-decorated dendrimer coated onto hydroxyapatite has been shown to enhance antibacterial activity [25].

Antimicrobial peptides (AMPs), typically consisting of 10–60 amino acids, have also been the focus of various studies developing new antimicrobial agents [26]. Almost all AMPs are amphipathic, allowing them to easily enter the cell membrane or pass through into the cytosol [27]. Lysine (K), arginine (R), histidine (H), leucine (L), and tryptophan (W) are also present in almost all AMPs [28,29,30]. The cationic characteristics of lysine and arginine and anionic characteristics of bacterial cell walls facilitate cell accessibility via electrostatic attraction, and the indole present in tryptophan drives the disintegration of bacterial cell walls. Additionally, studies have found that peptide sequences with cationic properties exhibited similar toxicity to cancer and bacterial cells. The (RW)_3_ peptide has exhibited particularly high antibacterial activity. The antibacterial mechanisms of AMPs depend on several physicochemical properties: charge, structure, sequence length, peptide concentration, hydrophobicity, and membrane composition [31].

Cathepsin B belongs to a family of lysosomal cysteine proteases known as cysteine cathepsins and plays an important role in intracellular proteolysis [32,33]. Cathepsin B is upregulated in certain cancers, in premalignant lesions, and in various other pathological conditions [34,35,36,37,38]. Additionally, cathepsins assume a significant part in bacterial microorganism killing [36]. The degradation of germs in the macrophage was aided by active cathepsins, such as cathepsin B, which served as a proteolytic or lipolytic agent [36]. Cathepsin B-sensitive peptides have been used to deliver anticancer genes or medications [39,40,41,42,43]. Among them, the Phe-Lys-Phe-Leu (FKFL) sequence was selected in this study as a potential AMP derivative when it was introduced multivalently at the peripheral ends of the PAMAM dendrimer [43]. Lysine’s cationic property makes it easier for the peptide to bind to the cell membrane [44]. It was also believed that leucine and phenylalanine would penetrate the membrane and disturb it [45]. Moreover, the cathepsin B enzyme may digest both bacteria and AMPs with FKFL, aiding in the preservation of healthy cells when it is activated in the macrophage during bacterial infection [36]. As a result, a cutting edge idea for treating bacterial infection is the use of the FKFL peptide in antibacterial compositions.

To this end, we have steered our efforts toward the development of amphiphilic dendrimers to address the critical need for new antibacterial agents for biomedical applications. In this study, we conjugated FKFL oligopeptides to the surface of the G2 PAMAM dendrimer (FKFL-G2) for an antibacterial for the first time. We investigated a straightforward and effective synthetic strategy to create a very effective antibacterial agent with a minimal toxicity on NIH3T3 cells. ^1^H NMR showed the overall synthesis yield was approximately 90%. The FKFL-G2 dendrimer can self-assemble because of its amphiphilic composition, and the critical aggregation concentration (CAC) was assessed (31.03 μM). The bacterial growth assay and colony-forming unit assay demonstrated the FKFL-G2 dendrimer inhibited the Gram-negative and Gram-positive bacterial strains. The mechanism of cell death was studied using the membrane permeabilization assay and FE-SEM. The data showed that the FKFL-G2 dendrimer disrupts the bacterial cell membrane, causing bacterial death. Therefore, our new material, namely the FKFL-G2 dendrimer, is promising for antibacterial applications in the biomedical field.

## 2. Materials and Methods

### 2.1. Materials

Polyamidoamine dendrimer ethylenediamine core generation 2 solution (G2 PAMAM), N,N-dimethylformamide (DMF), dimethylsulfoxide (DMSO), Fmoc-Leu-OH, Fmoc-Phe-OH, Fmoc-Lys (Boc)-OH, piperidine, triisopropylsilane (TIS), trifluoroaceticacid (TFA), diisopropylethylamine (DIPEA), dimethylsulfoxide (DMSO), deuterium oxide (D_2_O), Ampicillin, Kanamycin, n-phenyl-1-naphthylamine (NPN), and crystal violet were purchased from Sigma-Aldrich Korea (Seoul, Republic of Korea). 2-(1h-benzotriazole-1-yl)-1,1,3,3-tetramethyluronium hexafluorophosphate (HBTU) and hydroxybenzotriazole (HOBt) were purchased from Anaspec (San Jose, CA, USA). Paraformaldehyde (PFA) was purchased from Thermo Scientific and silicon wafers from TWOWIZ Wafermart. NIH3T3 cells were purchased from the Korean Cell Line Bank (Seoul, Republic of Korea).

### 2.2. Synthesis and Characterization of FKFL-G2 PAMAM Dendrimer

**Figure 1:**
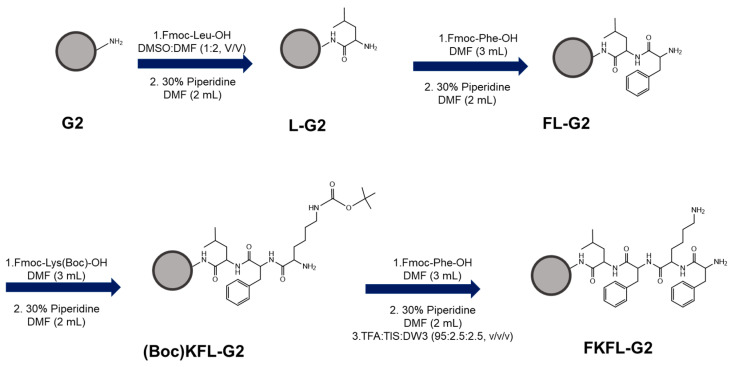
Schematic diagram of the synthesis of phenylalanine (F)-lysine (K)-phenylalanine (F)-Leucine (L)-polyamidoamine generation 2 (PAMAM G2) (FKFL-G2).

After being dried, 10 mg of PAMAM G2 (G2) was dissolved in anhydrous DMSO and DMF (*v*/*v*, 1:2). The solution was mixed with 4 equiv. of Fmoc-Leu-OH, HBTU, and HOBt and eight equiv. of DIPEA. The solution was stirred at 40 °C for 18 h. The resulting product was collected after precipitating in cold diethyl ether and washed three times. Deprotection was subsequently performed using 30% piperidine in DMF for 2 h at room temperature and confirmed using a ninhydrin test. Subsequent amino acid conjugation was carried out following the same procedure. The product was then dissolved in a mixture of TFA, TIS, and 3rd distilled water (DW3) (95:2.5:2.5, *v*/*v*/*v*) and reacted for 7 h to remove the tert-Butyloxycarbonyl-protecting group of Lys. Thereafter, the product was precipitated and washed with cold diethyl ether, dissolved in tertiary distilled water, and dialyzed in water for 24 h. The dialyzed product (FKFL-G2) was lyophilized, dissolved in D_2_O, and characterized using proton nuclear magnetic resonance (^1^H NMR) spectroscopy (600 MHz, AVANCE III 600, Bruker, Billerica, MA, USA). The overall synthesis scheme is shown in Figure 1.

### 2.3. Characterization of the FKFL-G2

#### 2.3.1. Mass Spectrometry

The FKFL-G2 dendrimer was dissolved in water at a concentration of 1 mg/mL. This sample was mixed with a 2,3-dihydroxybenzoic acid matrix; after which, the resulting mixture was placed on a plate and dried. Matrix-assisted laser desorption ionization time-of-flight mass spectrometry (MALDITOF MS) experiments were performed on a Bruker Autoflex Speed LRF spectrometer at the Mass Spectrometry Laboratory, University of Illinois.

#### 2.3.2. Critical Aggregation Concentration (CAC)

In order to confirm whether FKFL-G2 forms an aggregate by itself, the CAC was measured by fluorescence spectroscopy using Nile red. A 1 mL solution of FKFL-G2 with a concentration range of 0.001 to 0.9 mg/mL was combined with 5 µL of Nile red (1 mM in MeOH) and mixed by vortexing. Subsequently, fluorescence was measured at wavelengths of 550 nm for excitation and 560–760 nm for emission using a fluorometer (LS45, Fluorescence Spectrometer, PerkinElmer), and the fluorescence intensity at 650 nm was analyzed to measure the values of the nanoparticles.

#### 2.3.3. Zeta Potential and Size

The surface charge and the mean diameter of the FKFL-G2 were measured in triplicate and evaluated at various concentrations at room temperature using a Zetasizer Nano ZS (Malvern Panalytical, Malvern, UK).

### 2.4. Cytotoxicity Assay

NIH3T3 cells were used to evaluate the cytotoxicity of the FKFL-G2 dendrimer. A total of 1.8 × 10^4^ cells/well were seeded in a 96-well plate and incubated in 90 µL of Dulbecco’s modified Eagle’s medium, containing 10% (*v*/*v*) fetal bovine serum and 1% (*w*/*v*) penicillin/streptomycin, for 16 h at 37 °C in a humidified atmosphere containing 5% CO_2_. The cells were subjected to 10 µL of water (control) or 10 µL of polyethyleneimine (PEI 25 kDa) and G2 PAMAM solution, respectively. Each sample in the experimental group was treated with 10 µL of the FKFL-G2 dendrimer at the following concentrations: 12.5, 25, 50, 100, and 200 µg/mL. After 24 h of incubation, 10 µL of EZ-Cytox reagent (WST-8) was added to each well (except for that used as the negative control), which were incubated for a subsequent 2 h. Lastly, the absorbance of the wells measured a data wavelength of 450 nm via a colorimetric enzyme-linked immunosorbent assay (VERSAmax, Molecular Devices, Sunnyvale, CA, USA).

### 2.5. Bacterial Growth Assay

The antibacterial effect of FKFL-G2 was evaluated using *Escherichia coli* (*E. coli*) DH5α, which is kanamycin-resistant, and *Staphylococcus aureus* (*S. aureus*). Bacterial growth was estimated based on turbidity measurements, a commonly used method of evaluating the density of bacteria grown on a solution medium. In this process, *E. coli* and *S. aureus* bacteria were thawed, cultured overnight, and subcultured. Thereafter, 900 μL of each of these bacteria were incubated with 100 µL (100 µg) of the FKFL-G2 sample, as well as G2 PAMAM, kanamycin, and ampicillin, for 1, 2, 4, and 8 h while shaking the incubator at 225 rpm at 37 °C. The final concentration of all the reagents was set to 100 µg/mL (FKFL-G2 dendrimer, 8.46 µM; G2 PAMAM, 30.71 µM; kanamycin, 171.6 µM; and ampicillin, 286.2 µM, respectively) in the following experiments. The optical density (OD) of each sample was measured at a wavelength of 600 nm using an ultraviolet–visible spectrophotometer (OPTIZEN QX, Optizen, Daejeon, Republic of Korea) [46].

### 2.6. Colony-Forming Unit Assay

A colony-forming unit assay was performed to determine the effect of FKFL-G2 on bacterial colony formation. This process followed the same steps as the turbidity test in Section 2.5. The OD of the cells was measured after 24 h of growth. The cell solution was diluted by a factor of 10^7^; after which, 100 µL of the diluted solution was sprayed onto an agar plate and incubated in a shaking incubator for 24 h at 37 °C. The number of colonies produced was then observed.

### 2.7. Minimum Growth Inhibitory Concentration (MIC) Test

Absorbance was measured to determine the minimum growth inhibitory concentration of FKFL-G2. After culturing *E. coli* and *S. aureus* overnight in LB medium at 37 °C, some bacteria were transferred to a fresh medium and cultured further until the optical density (OD) reached 0.6. FKFL-G2 was treated to a bacterial solution at a final concentration of between 6.25 and 200 µg/mL (0.53 and 16.9 µM) and incubated for 2 h (for *E. coli*) or 8 h (for *S. aureus*) in a shaking incubator at 37 °C and 225 rpm. The absorbance was measured at a wavelength of 600 nm.

### 2.8. Membrane Permeabilization Assay

To evaluate the ability of FKFL-G2 to disrupt bacterial cell membranes, the membrane permeabilization assay was performed using N-phenyl-1-naphthylamine (NPN) [47]. *E. coli* and *S. aureus* bacteria were thawed and cultured overnight at 37 °C while being shaken at 180 rpm. After the absorbance of the bacterial solutions reached an OD_600_ of 1.0, the bacterial cells were harvested via centrifugation at 3500 rpm for 10 min. The bacterial pellet was washed twice with 4-(2-hydroxyethyl)-1-piperazineethanesulfonic acid (HEPES) buffer (pH 7.8). The bacterial pellets were then subjected to another round of centrifugation at 3500 rpm for 10 min and resuspended in 1 mL of HEPES (pH 7.8) to make bacterial solutions. Lastly, a 1 mL solution, containing 880 µL of a given bacterial solution; 20 µL of 1mM NPN; and 100 µL of FKFL-G2, G2 PAMAM, kanamycin, and ampicillin (1 mg/mL), was prepared and incubated for 30 min. Triton X-100, a well-known surfactant with a high capacity to destroy cell membranes, was used as a control. The fluorescence of each solution was measured using a fluorometer (LS45 Fluorescence Spectrometer, PerkinElmer, Waltham, MA, USA) at an excitation wavelength of 350 nm and emission wavelength of 429 nm.

### 2.9. Scanning Electron Microscopy

Bacterial morphology with untreated or Amp, Kan, G2, and FKFL-G2-treated was observed using field emission scanning electron microscopy (FE-SEM; S-4800, Hitachi, Tokyo, Japan). *E. coli* and *S. aureus* bacteria were cultured and incubated with experimental and control samples for 8 h at 37 °C while being shaken at 225 rpm. Thereafter, 50 µL of the bacterial solution of each culture was placed on a silicon wafer and dried at room temperature. The bacteria-containing wafers were placed on a 24-well plate and treated with 4% paraformaldehyde for 30 min. The surfaces were then washed using various concentrations of ethanol solution 0%, 25%, 50%, 75%, and 99.9%. The washed wafers were coated with platinum for 90 s; after which, and the appearance of bacteria was observed using FE-SEM (S-4800, Hitachi, Tokyo, Japan). The shapes of treated bacterial cells were compared with those of nontreated corresponding bacterial cells.

### 2.10. Biofilm Formation Assay

*E. coli* cells were thawed, cultured overnight, and then subcultured. Thereafter, 90 µL of *E. coli* cells (OD_600_ = 0.6) were incubated with 10 µL of either the FKFL-G2 samples or a mixture of G2 PAMAM, kanamycin, and ampicillin on a 96-well microplate for 8 h at 37 °C while being shaken at 225 rpm. To prevent subsequent background staining, the culture medium was removed and washed three times with distilled water. Then, 125 μL of crystal violet solution (0.1%, *w*/*v*) was added to the cells, which were incubated for another 10 min. Thereafter, the solution was removed, and the cells were washed with water and dried overnight. Lastly, 125 µL of a 30% acetic acid solution was added to the cells, which were incubated for 10 min to dissolve the film. The absorbance of the cells was then measured using a microplate reader (VERSAmax, Molecular Devices, Sunnyvale, CA, USA) at a wavelength of 550 nm.

### 2.11. Statistical Analysis

Statistical analysis of the experimental data was performed by the Student’s *t*-test using the GraphPad Prism 5 program (**, *p* < 0.01 and ***, *p* < 0.001).

## 3. Results

### 3.1. Characterization of the FKFL-G2 Dendrimer

**Figure 2:**
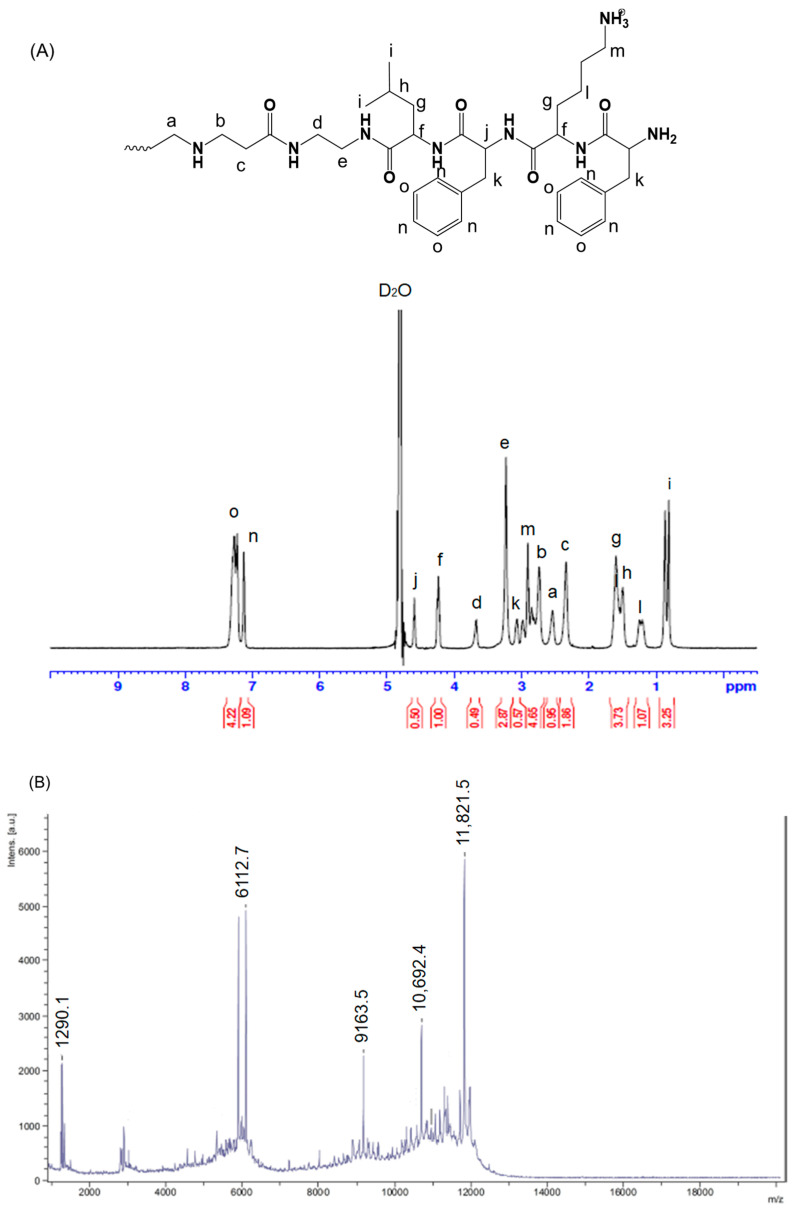

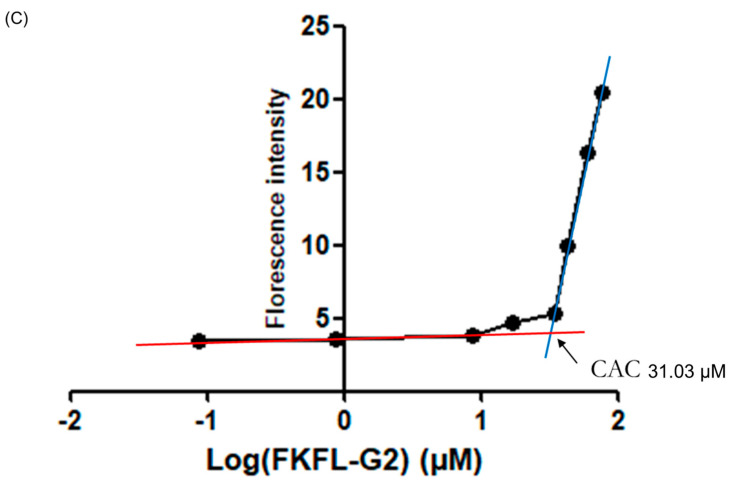
(**A**) Proton nuclear magnetic resonance data of FKFL-G2. (**B**) Matrix-assisted laser desorption/ionization–time-of-flight mass spectrometry data of FKFL-G2. (**C**) Critical aggregation concentration of FKFL-G2.

Following the steps outlined in Section 2.2, FKFL-G2 was successfully synthesized and analyzed using ^1^H NMR spectroscopy. The results (Figure 2A) showed that the conjugation yield of FKFL-G2 was 98%, 99%, and 90% for leucine, lysine, and phenylalanine, respectively. The final overall synthesis yield was approximately 90%.

FKFL-G2: δ (in ppm) 0.91 (-CHCH_2_CH(C**H**_3_)_2_-, leucine), 1.25 (-CHCH_2_C**H**_2_CH_2_CH_2_NH_2_, leucine), 1.49 (-CHCH_2_C**H**(CH_3_)_2_-, leucine), 1.76 (-CHC**H**_2_CH(CH_3_)_2_-, leucine; -CHC**H**_2_CH_2_CH_2_CH_2_NH_2_, lysine), 2.52 (-CONHCH_2_C**H**_2_N-, PAMAM), 2.83 (-NC**H**_2_CH_2_CO-, PAMAM), 2.86 (-CHCH_2_CH_2_CH_2_C**H**_2_NH_2_, lysine), 3.10 (C_6_H_5_C**H**_2_CH-,phenylalanine), 3.46 (-CONHCH_2_C**H**_2_NHCO, PAMAM), 3.66 (-CONHC**H**_2_CH_2_NHCO, PAMAM), G2), 3.95 (C_6_H_5_CH_2_C**H**-, phenylalanine), 4.3 (-C**H**CH_2_CH(CH_3_)_2_-, leucine), 4.53 (-C**H**CH_2_CH_2_CH_2_CH_2_NH_2_-, lysine), and 7.27 (C_6_**H**_5_CH_2_CH-, phenylalanine). As in Figure 2B, the molecular weight of FKFL-G2 was determined to be 11,821.5 g/mol using mass spectrometry (calculated: 11,826.28 g/mol based on ^1^HNMR). These results indicate the successful synthesis of FKFL-G2. It was also demonstrated that the FKFL-G2 dendrimer could self-assemble, forming a dendrimeric micelle at the concentration of 31.03 µM (0.415 mg/mL) (Figure 2C). Table 1 shows that the FKFL-G2 micelle has a nanometer size and a positive surface charge at above the CAC value. The findings showed that, as the FKFL-G2 dendrimer concentration increased, the surface zeta potential increased and micelle size reduced. This is a result of the hydrophobic F and L residues reducing the hydrophilicity of PAMAM G2 in an aqueous solution. A stronger hydrophobic interaction could result from an increase in internal hydrophobic density, which would result in a smaller size. Moreover, the enhanced primary amine groups from the higher concentrations of clustered FKFL-G2 are responsible for the micelles’ increased surface zeta potential.

### 3.2. Cytotoxicity Assay

**Figure 3:**
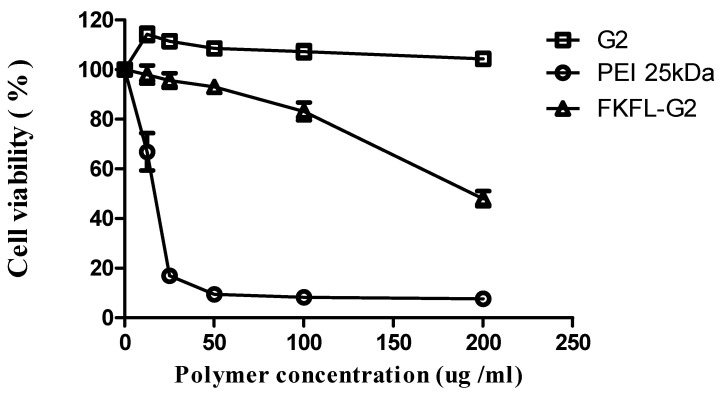
Cytotoxicity of FKFL-G2 in NIH3T3 cells, determined using an EZ-Cytox reagent assay. The data of the data points represent the means ± SD (*n* = 4).

As shown in Figure 3, PEI (25 kDa) was associated with a low cell viability in the NIH3T3 cell. This result is consistent with the previous study [48]. The findings of the cytotoxicity assay revealed that PEI (25 kDa) had substantial cytotoxicity and low cell viability in the cell line. G2 PAMAM, on the other hand, showed good biocompatibility, because it had a negligible impact on cell viability. At high doses, FKFL-G2 was largely nontoxic to noncancerous NIH3T3 cells, with the cell viability dropping from about 83% to 48% at 100 and 200 µg/mL (8.46 µM and 16.9 µM), respectively. It may need further research to find the right amount of FKFL-G2 to use as an antibiotic, balancing the drug’s potential toxicity to mammalian cells with its efficiency in treating bacterial infections.

### 3.3. Bacterial Growth Assay

**Figure 4:**
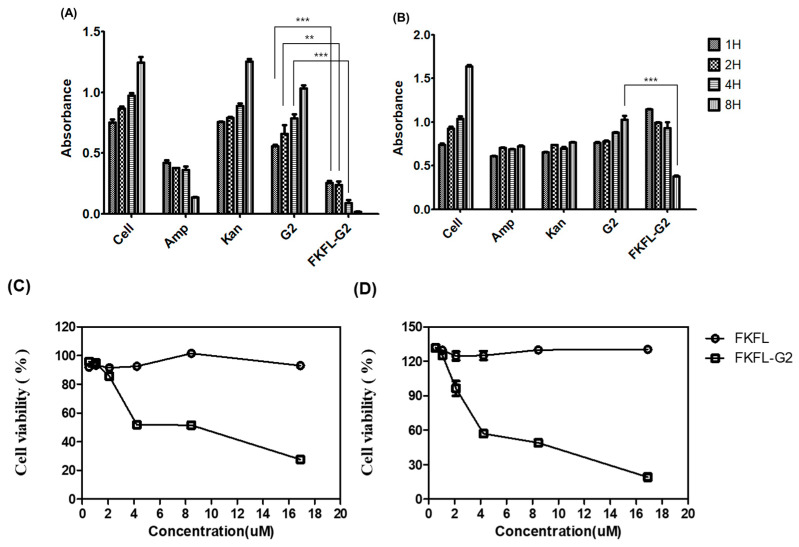
Antibacterial effect of FKFL-G2, second-generation polyamidoamine (G2 PAMAM), kanamycin (Kan), and ampicillin (Amp) on (**A**) *E. coli* and (**B**) *S. aureus*. The relationship between the FKFL peptide and FKFL-G2 concentration and bacterial cell viability was evaluated in (**C**) *E. coli* and (**D**) *S. aureus*. The results are shown as the mean ± SD (*n* = 3). Statistical analysis of the experimental data was performed by the Student’s *t*-test using the GraphPad Prism 5 program (**, *p* < 0.01 and ***, *p* < 0.001).

The bacterial growth assay is a popular method for determining the antibacterial activity of different substances [49,50]. The OD_600_ values were measured to analyze the proliferation of *E. coli* and *S. aureus* cells in the presence of ampicillin, kanamycin, G2 PAMAM dendrimer, and FKFL-G2. The results showed that *E. coli* cells grew well in the nontreated and kanamycin-treated samples, reflected in an increase in OD_600_ over time (Figure 4A). Ampicillin exhibited OD_600_ values of 0.423, 0.394, 0.361, and 0.137 after 1, 2, 4, and 8 h of incubation, respectively. Exhibiting low corresponding OD_600_ values of 0.254, 0.240, 0.091, and 0.017, FKFL-G2 had a higher antibacterial effect on *E. coli* than ampicillin. In the case of *S. aureus* (Figure 4B), the OD_600_ levels of the bacteria treated with ampicillin, kanamycin, G2 PAMAM, and FKFL-G2 for 1 h were similar to those of the nontreated bacteria. However, the OD_600_ of the nontreated bacterial cell solution increased over time, while the OD_600_ of the bacterial cell solution treated with ampicillin, kanamycin, and G2 PAMAM remained stable. These results suggest that ampicillin, kanamycin, and G2 PAMAM can inhibit *S. aureus* cell growth. Rather than remain stable, the OD_600_ of *S. aureus* cells treated with FKFL-G2 decreased over time; after 8 h, the OD_600_ of nontreated and FKFL-G2-treated *S. aureus* cells were 1.637 and 0.377, respectively. The results of the assay indicate that FKFL-G2 has a higher antibacterial efficacy than the antibiotics tested, as well as the G2 PAMAM dendrimer, showing that FKFL-G2 exhibited a more promising long-term reduction in cell growth. The MIC of FKFL-G2 was found to be between 5 and 8 μM (59.1 µg/mL and 94.56 µg/mL) for *E. coli* and 8 and 9 μM (94.56 µg/mL and 106.38 µg/mL) for *S. aureus*, respectively (Figure 4C). The results are significant, because they show that both Gram-positive and Gram-negative bacteria can be suppressed by FKFL-G2. The antibacterial impact of FKFL-G2 is thought to be caused not only by bacterial growth inhibition but also by the loss of membrane integrity. Therefore, additional experimentations have been conducted to back up this hypothesis regarding the FKFL-G2 action mechanism.

### 3.4. Colony-Forming Unit Assay

**Figure 5:**
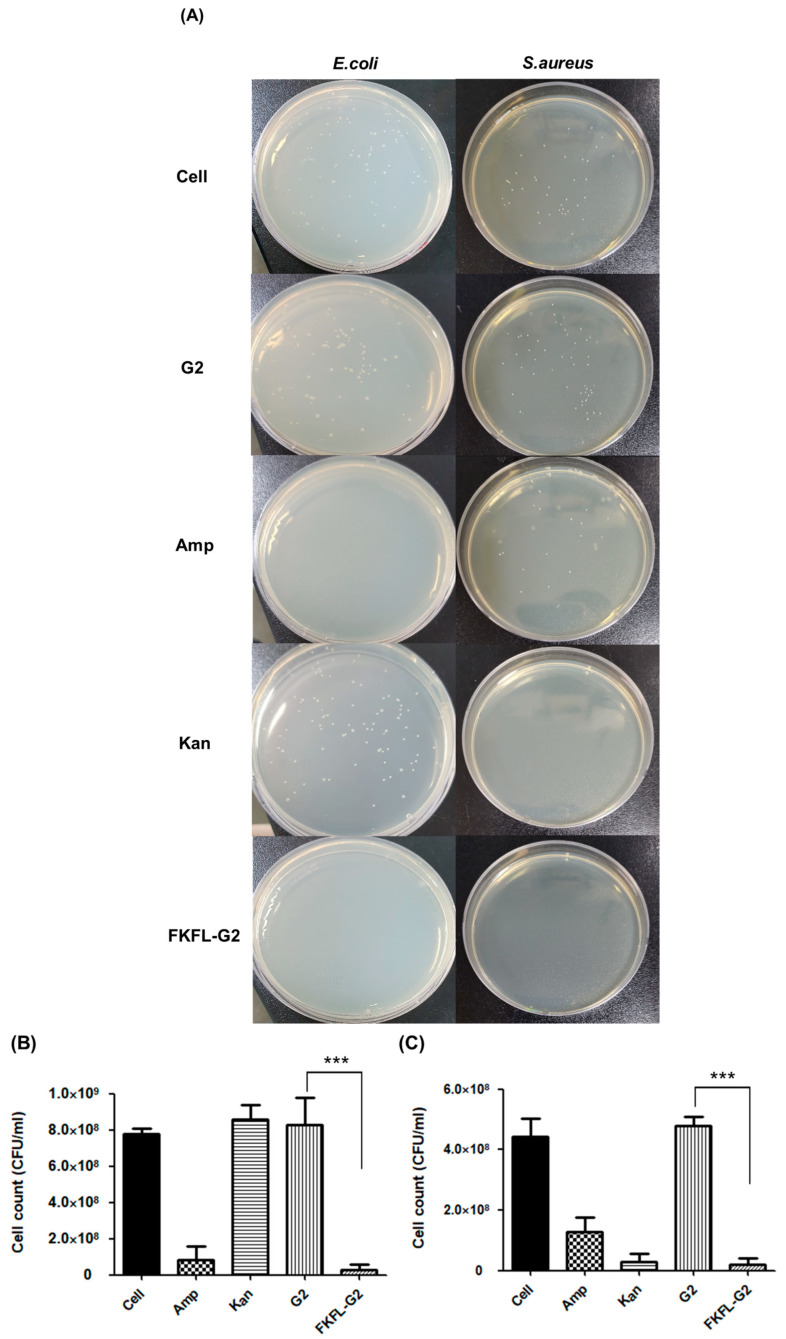
The effect of FKFL-G2, second-generation polyamidoamine (G2 PAMAM), kanamycin (Kan), and ampicillin (Amp) on bacterial colony formation. (**A**) Colony-forming unit assay images. The number of (**B**) *E. coli* and (**C**) *S. aureus* colonies. Results are presented as the mean ± SD (*n* = 3). Statistical analysis of the experimental data was performed by the Student’s *t*-test using the GraphPad Prism 5 program (***, *p* < 0.001).

G2 PAMAM and FKFL antibacterial G2 effectiveness against *E. coli* and *S. aureus* were assessed using the colony-forming unit (CFU) assay. Figure 5 shows the CFU counts of *E. coli* and *S. aureus* in all samples after 24 h of cell growth on an agar plate. Numerous colonies were observed in the PAMAM G2-treated samples, indicating that the G2 PAMAM dendrimer did not inhibit colony formation. In contrast, FKFL-G2 had a strong antibacterial effect on both *E. coli* and *S. aureus* cells. The findings of the CFU assay revealed that G2 PAMAM could not prevent colony growth, proving that it had little antibacterial power. The low CFU counts in the samples that had been exposed to FKFL-G2, on the other hand, showed that this substance had a potent antibacterial impact on both *E. coli* and *S. aureus* cells. According to this, the functionalization of the G2 PAMAM dendrimer with the FKFL peptide increases its antibacterial activity. Overall, the results of the CFU assay point to FKFL-G2 as a good target for additional antibacterial agent research.

### 3.5. Membrane Permeabilization Assay

**Figure 6:**
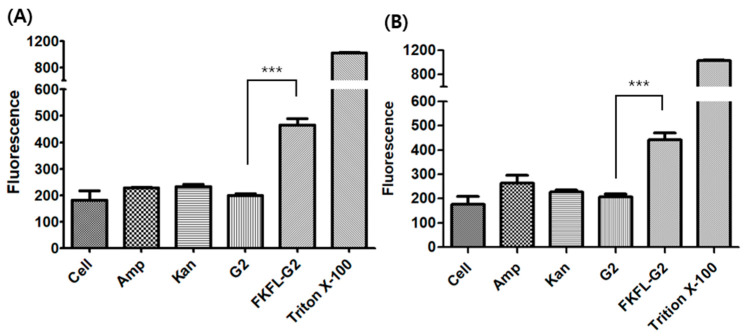
N-phenyl-1-naphthylamine assay results in illustrating the effect of TritonX-100, FKFL-G2, second-generation polyamidoamine (G2 PAMAM), kanamycin (Kan), and ampicillin (Amp) on the membrane of (**A**) *E. coli* and (**B**) *S. aureus*. Results are presented as the mean ± SD (*n* = 3). Statistical analysis of the experimental data was performed by the Student’s *t*-test using the GraphPad Prism 5 program (***, *p* < 0.001).

The capacity of antibacterial drugs to damage bacterial cell membranes can be determined using the membrane permeabilization assay [51]. FKFL-G2 was incubated with bacteria, and the effect of membrane permeabilization activity was evaluated by measuring the fluorescence of the cells treated with NPN. This fluorescent probe shows little intensity when it is unable to be incorporated into the intact lipid bilayer of bacteria, but it shows increased fluorescence according to the extent of the membrane destabilization, allowing the probe’s penetration into the membrane. As shown in Figure 6, Amp and Kan did not show any membrane perturbation effect, as expected. The native G2 PAMAM dendrimer showed no effect at all. However, in both the *E. coli* and *S. aureus* bacteria strains, FKFL-G2 exhibited higher fluorescence than PAMAM G2, which demonstrates that the antibacterial property of FKFL-G2 could be attributed to the membrane perturbation activity.

### 3.6. Scanning Electron Microscopy

**Figure 7:**
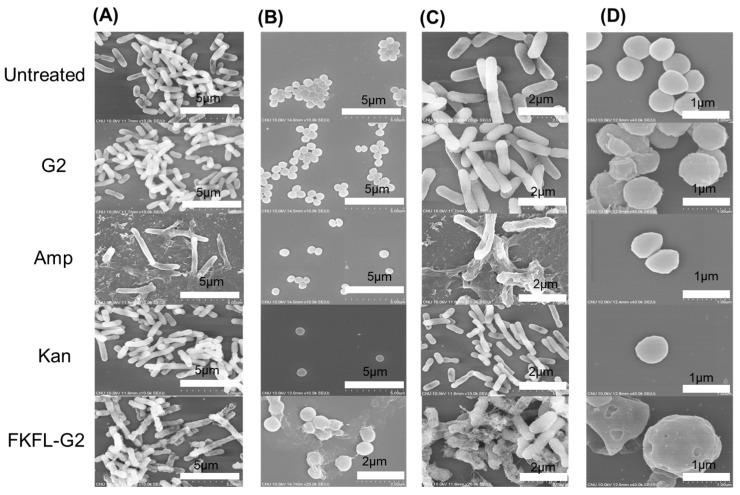
Bacterial morphology images of the untreated, G2-treated, Amp-treated, Kan-treated, and FKFL-G2-treated samples using field emission scanning electron microscopy at different magnifications. (**A**,**C**) *E. coli* and (**B**,**D**) *S. aureus* bacteria strains.

To further determine the antibacterial effect of FKFL-G2, SEM was performed. The visible evidence of FKFL antibacterial G2 action is provided by the SEM data. Figure 7 shows electron micrographs of treated and nontreated *E. coli* and *S. aureus* bacteria. The *E. coli* cells appeared rod-shaped, and *S. aureus* cells were circular. Both *E. coli* and *S. aureus* cell densities were lowest in the FKFL-G2-treated samples. The images reveal rough surfaces on the treated *E. coli* and *S. aureus* cells, which may be a result of FKFL-G2 harming the bacterial membrane.

### 3.7. Biofilm Formation Assay

**Figure 8:**
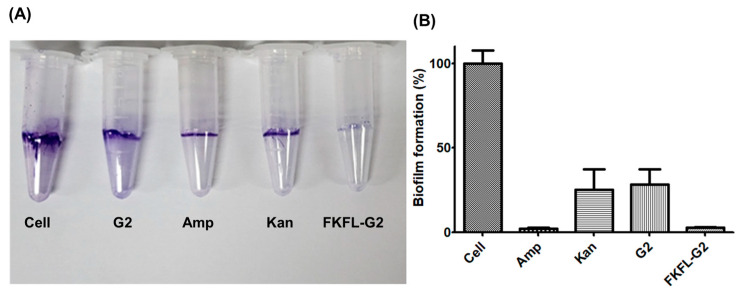
Biofilm assay results illustrating the effect of FKFL-G2, second-generation polyamidoamine (G2 PAMAM), kanamycin (Kan), and ampicillin (Amp) on *E. coli* biofilm formation. (**A**) Images of treated cells. (**B**) Absorbance data. Results are presented as the mean ± SD (*n* = 4).

Biofilms promote persistent bacterial infections and bacterial defenses against external threats [52]. Thus, a biofilm assay was performed to determine the effect of FKFL-G2 on biofilm formation as an additional facet of antibacterial activity. This experiment was conducted using crystal violet [53]. As shown in Figure 8, FKFL-G2 and ampicillin exhibited similarly high biofilm formation inhibition rates of approximately 98%, followed by kanamycin at 75% (Figure 8B). The biofilm formation inhibition effect of G2 PAMAM (Figure 8B) is possibly due to its amine group, which adheres to the bacterial cell wall via electrostatic attraction and prevents bacterial aggregation and colony formation (Figure 8A).

## 4. Discussion

The FKFL-G2 dendrimer was successfully synthesized with a high conjugation yield, as demonstrated by the ^1^H NMR and mass spectroscopy data. Since it contains multivalent amphiphilic peptides, FKFL-G2 can form dendrimeric micelles at a concentration of 31.03 µM (0.415 mg/mL). In addition, the primary amines of Lys and terminal ends of the peptides contribute to the positive charge of the micelles. A combination of basic amino acids with hydrophobic amino acids is also a general approach for designing antibacterial polymers [43,44,45].

Biocompatibility is one of the key issues facing the biomedical application of PAMAM dendrimers [54]. We used FKFL-G2 at the concentrations under CAC for biological tests. We hypothesized that the monomer itself could interact with bacterial cell membranes through electrostatic and hydrophobic interactions. As shown in Section 3.2, G2 PAMAM was confirmed to be safe for NIH3T3 cells, reflecting its status as a biocompatible material. High cell viability in the range of approximately 83% was observed in NIH3T3 cells treated with native dendrimers and FKFL-G2 at a concentration lower than 100 μg/mL (8.46 µM for FKFL-G2). This result confirms that FKFL peptide conjugation did not significantly alter the cytotoxicity of the original G2 PAMAM dendrimer at a concentration lower than 100 μg/mL in NIH3T3 cells. Additionally, the fact that FKFL-G2 dendrimers caused less cell death in the noncancerous fibroblast cells demonstrates that FKFL-G2 is a promising candidate for biomedical applications.

As demonstrated in Section 3.3 and Section 3.4, FKFL-G2 could inhibit the growth of both *E. coli* and *S. aureus* bacteria. The OD_600_ values of the FKFL-G2-treated samples were similar to those of the antibiotics-treated samples (ampicillin in the case of *E. coli* and both ampicillin and kanamycin in the case of *S. aureus*). In terms of its antibacterial mechanism, Section 3.5 and Section 3.6 indicated that FKFL-G2 interacted with the bacterial cell membrane via electrostatic binding. Many AMP contain lysine and arginine residues because of their capacity to bind to cell membranes [28,29,30]. By interacting with the cell membrane, FKFL-G2 disrupts the bacterial membrane integrity and ultimately leads to bacterial cell death. These discoveries make it clear how crucial it is to comprehend how antimicrobial substances interact with bacterial membranes and offer essential information on the mechanism of action of FKFL-G2. Further investigations could look into how the shape of the dendrimer impacts its interaction with cell membranes and how electrostatic interactions between FKFL-G2 and bacterial membranes affect its antibacterial activity.

Membrane penetration was demonstrated using NPN [43]. Both the Gram-negative *E. coli* and Gram-positive *S. aureus* cells treated with FKFL-G2 exhibited the highest fluorescence. The cell walls of the Gram-negative and Gram-positive cells differ; however, both have a negatively charged amphiphilic component [55]. The previous review article stated that the physicochemical properties of the surface exposed to a virus, such as the charge or hydrophobic/hydrophilic of the material, can affect nonspecific virus deposition [56]. Similarly, we hypothesize that the physicochemical properties of the material may also influence the structural destruction of the bacterial cell membrane. Both the positive surface charge and the hydrophobic feature from the FKFL peptides contribute to the interaction and disruption of the bacterial membrane. FKFL-G2 can easily interact with and penetrate the cell wall and membrane of both bacteria. One of the main components of the antibacterial peptide sequence is hydrophobic. Thus, the membrane of *S. aureus* may also have been damaged via its interaction with the hydrophobic component of FKFL-G2. The outcomes of this test demonstrate the antibacterial activity of FKFL-G2, as it was able to disrupt membranes and increase NPN fluorescence in both *E. coli* and *S. aureus* cell strains. This implies that FKFL-G2 has the potential to weaken bacterial cell membranes, causing them to become more permeable and ultimately die. However, the native G2 PAMAM dendrimer had no impact on the permeabilization of the membrane. This indicates that the precise chemical alterations introduced into the dendrimer, rather than just the presence of the G2 PAMAM dendrimer, are what give FKFL-G2 its antibacterial properties. Overall, these results provide further evidence for the antibacterial activity of FKFL-G2 and its potential as a novel antibacterial agent.

In addition, Figure 7 indicates that the treated cells showed damaged and perforated structures, which were not present in the untreated cells. These findings corroborate earlier findings, which demonstrated that FKFL-G2 has a significant impact on the bacterial membrane and can prevent their growth. One of the primary actions in which FKFL-G2 exerts its antimicrobial effects is by interacting with bacterial membranes and destabilizing them. The exact mode of action of FKFL-G2 needs to be studied further, but it is believed from the results that FKFL-G2 can interact with bacterial membranes by electrostatic interactions between the positively charged peripheral multivalent FKFL peptides and the negatively charged bacterial membranes. This can lead to a disruption of the membrane’s integrity and leakage of cellular components, which ultimately results in bacterial cell death. FKFL-G2 might also penetrate into the membrane, forming pores and causing disturbances in the membrane structure. The SEM results provide a clear and direct visualization of the morphological changes in the bacterial cells, confirming the effectiveness of FKFL-G2 as an antibacterial agent.

Moreover, the results of the biofilm assay suggest that FKFL-G2 has a strong inhibitory effect on biofilm formation (Figure 8), which is consistent with its antibacterial activity, as shown in previous assays. Biofilms are known to promote bacterial persistence and resistance to external threats such as antibiotics, making them a significant challenge in the treatment of bacterial infections. Therefore, the ability of FKFL-G2 to inhibit biofilm formation is a promising characteristic that could be useful in combating persistent infections.

In summary, the study shows that FKFL-G2 has promising antibacterial properties and suggests that it could be developed into a novel antimicrobial agent to combat bacterial infections. Future research could focus on investigating the broader spectrum of activity of FKFL-G2 against different bacterial strains and exploring its potential in combination therapy with other antibiotics. The potential cytotoxicity of FKFL-G2 toward mammalian cells and its potential in vivo effectiveness in animal models of bacterial infections should also be investigated in more investigations. These investigations will aid in determining the safety and efficacy of FKFL-G2 dendrimers for clinical application. Nevertheless, these encouraging findings offer a solid framework for further research and indicate that FKFL-G2 dendrimers may be useful in a range of medicinal applications.

## 5. Conclusions

In this study, a novel hybrid peptide-conjugated polymer was synthesized by combining the FKFL oligo peptide sequence with the G2 PAMAM dendrimer FKFL-G2. The method of synthesis proved simple and resulted in a high conjugation yield of the product. Additionally, FKFL-G2 exhibited low toxicity to noncancerous cells at concentrations up to 100 µg/mL (8.46 µM). Compared to Amp and Kan antibiotics, FKFL-G2 had either stronger or similar antibacterial effects on Gram-negative *E. coli* and Gram-positive *S. aureus* bacteria. The antibacterial effect of FKFL-G2 was due to its ability to disrupt the bacterial cell membrane. Therefore, FKFL-G2 shows potential in a new nanomaterial-based antibiotic development and applications.

## Figures and Tables

**Table 1:** Zeta potential and mean diameter values of FKFL-G2.

Concentration (µM)	Zeta Potential	Diameter ^a^	Polydispersity (PDI) ^a^
42.3	29.63 ± 6.51	158.33 ± 12.59	0.628 ± 0.14
84.6	35.5 ± 6.0	109.83 ± 17.54	0.459 ± 0.18
169.2	38.23 ± 5.74	98.45 ± 6.95	0.475 ± 0.03

^a^ Determined by dynamic light scattering (DLS) measurements at room temperature (RT); measurements were repeated three times.

## Data Availability

Data are contained within the article.
